# Prevalence and Impact of Pulmonary Hypertension Associated with Arteriovenous Fistulas and Grafts in End-Stage Renal Disease: A Systematic Review and Meta-Analysis

**DOI:** 10.3390/arm94040046

**Published:** 2026-07-06

**Authors:** Ahmed A. Zayed, Mohammad Aldalahmeh, Salim Barakat, Georges Khattar, Walid Sange, Elie Bou Sanayeh, Zaid Khamis, Bahy Abofrekha, Suzanne El-Sayegh, Michel N. Chalhoub

**Affiliations:** Department of Internal Medicine, Northwell Health, Staten Island, NY 10305, USA; maldalahmed@northwell.edu (M.A.);

**Keywords:** pulmonary hypertension, arteriovenous fistula, arteriovenous graft, end-stage renal disease, hemodialysis, vascular access, meta-analysis

## Abstract

**Highlights:**

**What are the main findings?**

- In a pooled analysis of observational studies, AVF/AVG use in dialysis patients was associated with higher odds of pulmonary hypertension than non-AVF/AVG access (pooled OR 2.06, 95% CI: 1.69–2.52). This association was statistically significant but sensitive to individual influential studies, and the low between-study heterogeneity (I^2^ = 0%) reflects directional consistency rather than robustness of the effect.
- Across the five studies reporting continuous data, mean pulmonary artery pressure tended to be higher in AVF/AVG than in non-AVF/AVG patients; however, extreme between-study heterogeneity (I^2^ = 99.4%) precluded a valid pooled estimate, so this outcome is described qualitatively rather than meta-analyzed.

**What are the implications of the main findings?**

- These findings may support consideration of echocardiographic monitoring for pulmonary hypertension in long-term hemodialysis patients, particularly those with high-flow brachial AVFs or prolonged dialysis vintage, but this should be regarded as a hypothesis to be tested prospectively rather than an established recommendation.
- Because the available evidence is observational and cannot establish causality, prospective hemodynamic studies are needed before access-planning strategies—such as flow reduction or alternative dialysis modalities—can be recommended for higher-risk patients.

**Abstract:**

Background/Objectives: Pulmonary hypertension (PH) is an increasingly recognized complication in patients with end-stage renal disease (ESRD) undergoing hemodialysis, particularly those utilizing arteriovenous fistulas (AVF) or grafts (AVG) for vascular access. The prevalence and clinical impact of PH in this population remain unclear due to methodological heterogeneity and variable diagnostic criteria. This systematic review and meta-analysis aimed to quantify the association between AVF/AVG use and PH prevalence in ESRD patients and to explore sources of heterogeneity. Methods: A systematic search of PubMed, Embase, Scopus, and Web of Science was conducted for studies published through 31 December 2024, without language or date restrictions. Eligible studies included adults (≥18 years) with ESRD on dialysis, comparing those with AVF/AVG access to non-AVF/AVG controls (e.g., tunneled dialysis catheters or peritoneal dialysis), and reporting PH prevalence or mean pulmonary artery pressures. Study quality was assessed using the Newcastle–Ottawa Scale, and risk of bias was evaluated. A random-effects meta-analysis calculated pooled odds ratios (OR) for PH prevalence, with heterogeneity assessed by I^2^ and Cochran’s Q. Sensitivity analyses and tests for publication bias (Egger’s and Begg’s) were performed. Secondary analysis compared pooled mean pulmonary artery pressures between groups. Results: Eleven observational studies (1299 dialysis patients) met the inclusion criteria; ten studies (1224 patients) contributed to the quantitative meta-analysis after exclusion of one study with a zero-event control arm. Most studies were small, predominantly cross-sectional, and of moderate methodological quality. The pooled analysis showed a statistically significant association between AVF/AVG use and PH (OR 2.06, 95% CI: 1.69–2.52), with low statistical heterogeneity (I^2^ = 0%). This estimate was sensitive to individual studies: in leave-one-out analysis the association lost statistical significance when the single most influential study was removed indicating that the pooled result is driven in part by a small number of studies rather than being uniformly robust. No statistical evidence of publication bias was detected. Five studies reported continuous pulmonary artery pressures, which were directionally higher in AVF/AVG patients but were not pooled because of extreme heterogeneity (I^2^ = 99.4%). Conclusions: In this synthesis of observational data, AVF/AVG use was associated with higher odds of pulmonary hypertension than non-AVF/AVG access. Because all included studies were observational and the pooled estimate is sensitive to individual influential studies, these findings indicate a possible association rather than a causal effect and should be interpreted with caution. They support the rationale for prospective hemodynamic studies and for evaluating—rather than presuming the benefit of—PH monitoring and individualized access strategies in higher-risk dialysis patients.

## 1. Introduction

End-stage renal disease (ESRD) requires renal replacement therapy, with hemodialysis being the primary modality for over two million patients worldwide [1]. Arteriovenous fistulas (AVFs) and grafts (AVGs) are the preferred vascular access types due to superior long-term patency and lower infection rates compared to central venous catheters [2]. However, pulmonary hypertension (PH)—defined by elevated pulmonary arterial pressures—has emerged as a significant and underrecognized complication in this population, with reported prevalence rates ranging from 17% to 70% [1,3,4].

PH in patients with ESRD is a complex, multifactorial condition driven by a constellation of hemodynamic, biochemical, and structural alterations [2,3]. Hemodialysis via AVF contributes to this pathophysiology through sustained high-flow states that can increase cardiac output by up to 40%, thereby overwhelming pulmonary vascular compliance and promoting vascular remodeling [3]. Concurrently, chronic volume overload, uremia-induced endothelial dysfunction, increased oxidative stress, and diminished nitric oxide bioavailability contribute to elevated pulmonary vascular resistance, perpetuating a maladaptive cardiopulmonary feedback loop [3]. These processes collectively enhance right ventricular afterload and compromise cardiovascular function. Notably, PH in this population has been independently associated with a twofold increase in all-cause mortality [1,5].

Despite its clinical relevance, reported PH prevalence among dialysis patients varies widely. Echocardiographic assessments indicate a median prevalence of approximately 38% in hemodialysis populations, compared to 19% in those undergoing peritoneal dialysis (PD), reflecting potential modality-related pathophysiological differences [1]. However, this variation is further confounded by methodological heterogeneity, including inconsistencies in diagnostic criteria (e.g., reliance on echocardiography versus right heart catheterization), differences in AVF/AVG flow characteristics, and variable dialysis vintage [4]. Interdialytic fluid shifts and fluctuations in intravascular volume status also complicate the accuracy and timing of pulmonary pressure measurements [3,4].

Current literature is limited by a lack of focused, synthesized evaluation of the specific contribution of AVF/AVG access to PH development. While some studies suggest that access-related hemodynamics play a causative role, others posit that AVFs merely reveal preexisting subclinical cardiopulmonary disease [2,6]. To clarify these associations, this meta-analysis systematically quantifies the relationship between AVF/AVG use and PH prevalence in ESRD populations, while exploring underlying sources of heterogeneity. By consolidating evidence from diverse study populations, this analysis aims to inform risk stratification, vascular access planning, and routine screening protocols to improve long-term outcomes in patients undergoing maintenance dialysis.

## 2. Materials and Methods

This systematic review and meta-analysis was conducted in accordance with the Preferred Reporting Items for Systematic Reviews and Meta-Analyses (PRISMA) guidelines (Supplementary Material). The study protocol was prospectively registered in the International Prospective Register of Systematic Reviews (PROSPERO) under the registration number CRD420251051561.

### 2.1. Search Strategy

A comprehensive and systematic literature search was performed across PubMed, Embase, Scopus, and Web of Science, covering all publications from database inception through 31 December 2024. No language or publication date restrictions were imposed to enhance inclusivity and reduce selection bias. To ensure completeness, reference lists of included studies and relevant systematic reviews were manually screened for additional eligible records.

The search strategy incorporated both Medical Subject Headings (MeSH) and relevant keywords. The following Boolean combination was used:

(“Renal Dialysis”[MeSH] OR “End-Stage Renal Disease”[MeSH] OR “ESRD”) AND (“Arteriovenous Fistula”[MeSH] OR “AVF” OR “Vascular Access”) AND (“Hypertension, Pulmonary”[MeSH] OR “Pulmonary Hypertension” OR “Pulmonary Artery Pressure” OR “RVSP” OR “PASP”).

Boolean operators “AND” and “OR” were used to maximize the sensitivity of the search strategy. No restrictions were applied regarding the nature of the comparator or outcome variables to allow for broad capture of relevant studies. All retrieved citations were imported into Covidence for systematic screening, where duplicate entries were removed prior to title and abstract screening.

### 2.2. Eligibility Criteria

Inclusion criteria:

- Population: Adult patients (≥18 years) with ESRD undergoing dialysis.
- Intervention: Presence of an AVF OR AVG for dialysis access.
- Comparison: Patients without AVF, including those with tunneled dialysis catheters (TDC), PD, or conservative management.
- Outcomes: Studies reporting on the prevalence of PH or mean pulmonary artery pressures (PAP, mPAP, PASP, RVSP).
- Study design: Observational studies (prospective or retrospective cohorts, cross-sectional, case–control) and clinical trials.

Exclusion criteria:

- Studies involving patients < 18 years of age.
- Reviews, editorials, commentaries, or non-peer-reviewed sources.
- Studies that did not include a comparator group (e.g., no non-AVF/AVG control).
- Studies that did not report either prevalence or continuous PH metrics.
- Duplicate studies or those with overlapping populations (the most complete version was retained).

### 2.3. Data Extraction and Quality Assessment

Three reviewers (MA, SB, and EB) independently extracted data from each eligible study using a pre-defined, standardized data collection form. Extracted variables included: first author, year of publication, study design, patient population characteristics, total sample size, number of patients with AVF/AVG and non-AVF/AVG access, definition and diagnostic method of pulmonary hypertension (PH), and the number of PH cases within each access group. When PH prevalence was reported as a percentage, the absolute number of cases was calculated based on the corresponding group sample size. To assess methodological quality, study-specific risk of bias tools were employed. For observational studies, the Newcastle–Ottawa Scale (NOS) was used to evaluate three domains: selection of study groups, comparability of cohorts, and ascertainment of outcomes. For randomized controlled trials (RCTs), the Cochrane Risk of Bias 2.0 tool was applied. Any discrepancies in data extraction or quality assessment were resolved by consensus or, when necessary, through adjudication by a fourth reviewer (GK).

### 2.4. Statistical Analysis

The primary outcome was the association between AVF/AVG access and PH, expressed as an odds ratio (OR) with corresponding 95% confidence intervals (CIs). Study-specific ORs were calculated from 2 × 2 contingency tables comparing PH presence in AVF/AVG vs. non-AVF/AVG groups.

A random-effects model using the DerSimonian–Laird method was chosen for meta-analysis to account for expected between-study heterogeneity. The pooled OR and CI were estimated using log-transformed ORs and standard errors. Statistical significance was set at a *p*-value < 0.05.

Heterogeneity was assessed using Cochran’s Q test and I^2^ statistic. I^2^ values were interpreted as follows: 0–30% (low heterogeneity), 30–50% (moderate), 50–75% (substantial), and >75% (considerable heterogeneity). A Q-test *p*-value < 0.10 was considered indicative of significant heterogeneity. To assess the robustness of the pooled estimate, a leave-one-out sensitivity analysis was conducted. The meta-analysis was recalculated iteratively after removing one study at a time to determine the influence of individual studies on the overall result. Special attention was paid to high-impact studies such as Fabbian et al., whose removal led to notable attenuation of effect size. Publication bias was assessed using two statistical tests: Egger’s regression test and Begg’s rank correlation test. Given that the number of included studies was limited to 10, visual interpretation of funnel plot asymmetry was considered unreliable and not used as a standalone method. Egger’s test evaluates funnel plot asymmetry by regressing the standardized effect sizes against the inverse of their standard errors (precision). This method is sensitive to small-study effects but may lack power with a small number of studies. To complement this, Begg’s test, a non-parametric rank correlation method based on Kendall’s tau, was used due to its greater suitability in meta-analyses with fewer than 10–15 studies. Together, these tests provided a statistically robust approach to identifying potential publication bias despite the limited sample of included studies. As a secondary analysis, we also compared mean pulmonary artery pressure (PAP) values between AVF/AVG and non-AVF/AVG groups across a subset of studies that reported continuous data. A random-effects model was used to estimate the pooled mean difference in PAP. Given the expected variability in measurement and populations, heterogeneity was assessed using the Q statistic and I^2^. All analyses were conducted using Python version 3.8. The “statsmodels”, “scipy”, and “matplotlib” libraries were used for effect size computation, statistical testing, and visualization.

## 3. Results

**Figure 1:**
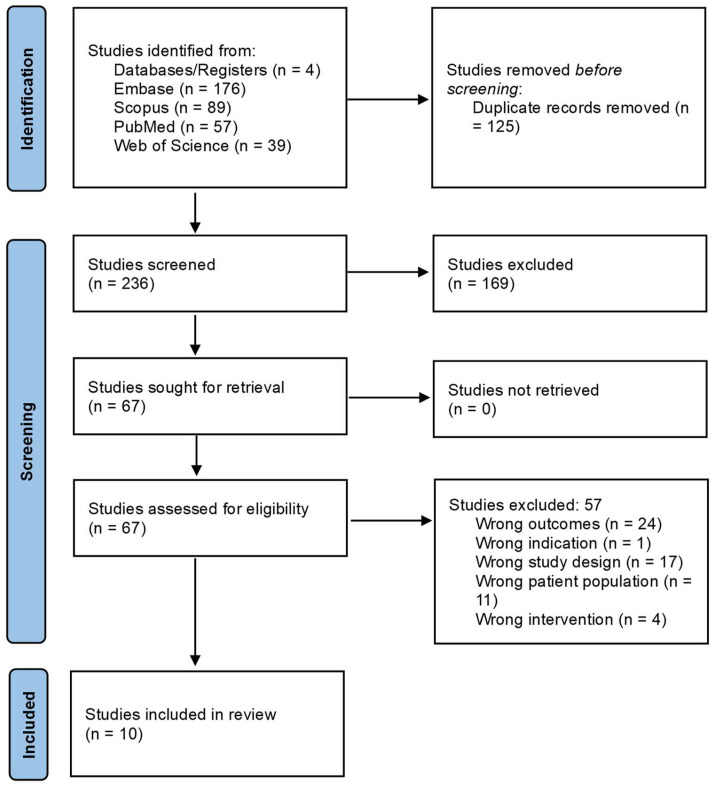
PRISMA Flow Diagram of Study Selection Process.

Figure 1 presents the PRISMA diagram, illustrating the number of studies included at each stage of the evaluation process. A total of eleven observational studies (1299 dialysis patients) met the inclusion criteria for the systematic review. Ten studies (1224 patients) were included in the quantitative meta-analysis after exclusion of one study with a zero-event control arm (detailed below). Table 1 summarizes the studies comparing patients with AVF/AVG access to those with alternative or no access types, including TDC, PD, or pre/post-AVF creation. The primary outcome was the presence of PH, defined variably across studies as elevated pulmonary artery pressure (PAP), right ventricular systolic pressure (RVSP), or mean PAP via echocardiography or right heart catheterization.

The quality assessment of the 10 included studies using the NOS showed that most were of moderate methodological quality, with total scores ranging from 5 to 7 out of a possible 9. Common strengths included representative patient cohorts, clear documentation of exposure and outcome, and, in several studies, careful exclusion of major confounders. However, limitations were also apparent: most studies employed cross-sectional designs, lacked baseline PH assessment prior to AVF creation or dialysis initiation, and did not fully adjust for all relevant confounders. Only a few studies have reached a good quality rating, typically due to more comprehensive adjustment for confounders or the use of longitudinal data. Overall, while the evidence base benefits from standardized outcome assessment and representative sampling, the moderate quality of most studies highlights the need for cautious interpretation of causal relationships between arteriovenous fistula use and PH. Detailed NOS scoring for each study is provided in the Supplementary Material.

Yigla et al. reported no pulmonary hypertension events in the comparator arm, yielding an unstable odds ratio (22.34, 95% CI: 1.28–390.55) whose interval spans more than two orders of magnitude. Because this zero-event configuration makes the study estimate highly dependent on the choice of continuity correction and exerts disproportionate influence within a pool of otherwise modestly sized studies, it was not included in the primary quantitative synthesis; its reported estimate is nonetheless presented in Table 1 for transparency. We acknowledge that excluding an influential early study is itself a limitation, and that methods incorporating it through a continuity correction could yield a different pooled estimate (see Limitations).

**Figure 2:**
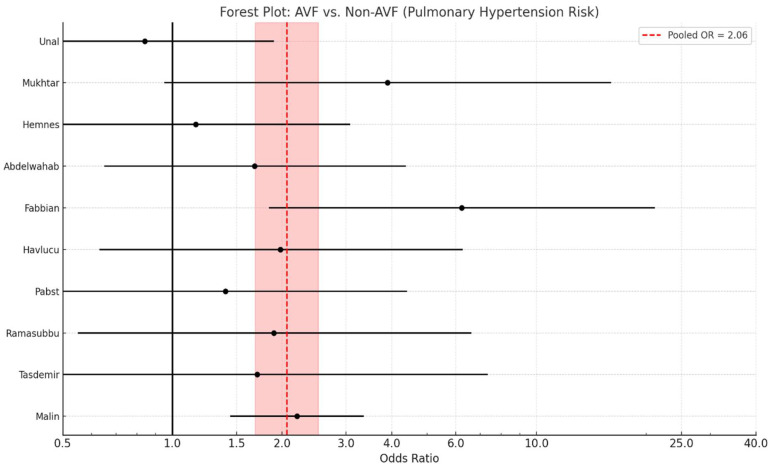
Forest Plot of Odds Ratios Comparing AVF vs. Non-AVF in PH Risk.

The pooled analysis, using a random-effects model, demonstrated a significant association between AVF and PH, with a pooled OR of 2.06 (95% CI: 1.69–2.52), as shown in Figure 2.

Statistical heterogeneity was low (Q statistic = 5.37, df = 9, *p* = 0.80; I^2^ = 0%). A low I^2^ in a synthesis of small studies with wide confidence intervals reflects consistency in the direction of effect and limited power to detect heterogeneity, rather than robustness of the pooled estimate—which, as shown above, is influenced by individual studies.

A leave-one-out sensitivity analysis (Table 2) showed that the pooled estimate was sensitive to individual studies. Exclusion of Fabbian et al. alone attenuated the pooled OR to 1.19 (95% CI: 0.81–1.75), rendering the association statistically non-significant, whereas exclusion of Unal et al. yielded an OR of 1.74 (95% CI: 0.97–3.11); removal of the remaining studies left the estimate broadly unchanged. The loss of statistical significance upon removal of a single moderate-quality study indicates that the pooled association is driven in part by a small number of influential studies and should therefore be interpreted as a possible, rather than firmly established, effect.

**Figure 3:**
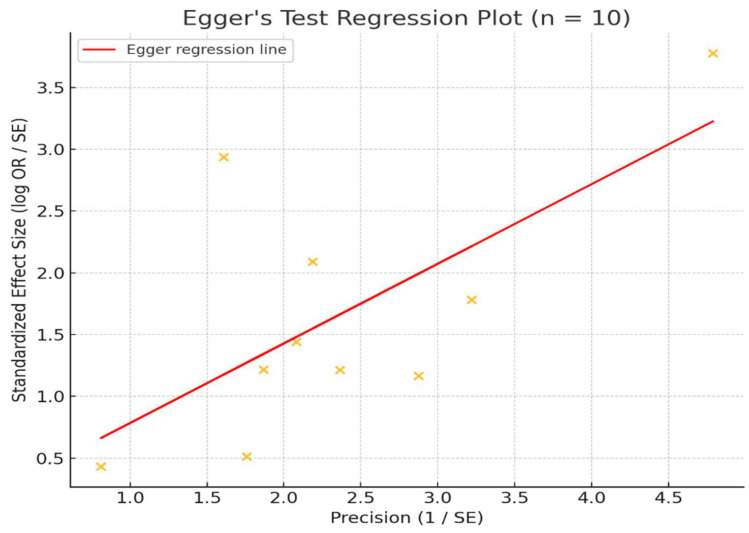
Egger’s regression test for funnel-plot asymmetry. Each × denotes one of the ten studies in the quantitative meta-analysis.

**Figure 4:**
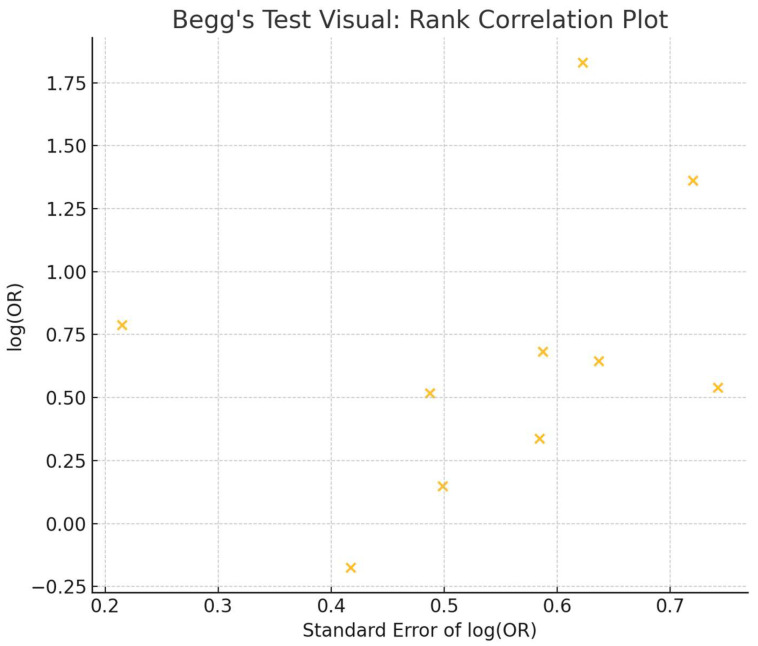
Funnel plot underlying Begg’s rank-correlation test for publication bias. Each × denotes one of the ten studies in the quantitative meta-analysis.

There was no evidence of publication bias based on formal statistical tests. Figure 3 and Figure 4 show, respectively, the results of Egger’s regression test—yielding an intercept of 0.14 (*p* = 0.83)—and Begg’s rank correlation test, which produced a Kendall’s tau of 0.29 (*p* = 0.29). These findings suggest that the pooled effect is unlikely to be influenced by small-study effects or selective reporting.

Five studies reported mean pulmonary artery pressures separately for AVF/AVG and non-AVF/AVG groups. Although values were directionally higher in the AVF/AVG group across these studies, the between-study heterogeneity was extreme (I^2^ = 99.4%), indicating that almost all observed variation reflected true differences between studies rather than sampling error. A pooled mean difference under these conditions would represent an average of non-comparable quantities; it is therefore not reported as a quantitative finding, and this secondary outcome is described qualitatively only.

## 4. Discussion

Our meta-analysis identified a statistically significant association between AVF/AVG use and PH in dialysis patients (pooled OR 2.06, 95% CI: 1.69–2.52), with low statistical heterogeneity. As detailed below, however, this estimate was sensitive to individual studies, and the low I^2^ reflects consistency in the direction of effect rather than robustness of its magnitude; the association should therefore be regarded as suggestive rather than confirmatory.

These findings are consistent with mechanistic studies linking AVF-associated hemodynamic changes to PH development. Elevated pulmonary vascular resistance (PVR) and HD duration have been proposed as critical mediators [18]. Furthermore, brachial AVF location—associated with higher flow rates—was reported to show a 100% PH prevalence in one study, compared to 66.7% in radial AVF patients, although this disparity requires validation in larger populations [4,18].

Clinically, the heightened PH risk in AVF users intersects with mortality data: ESRD patients with echocardiographic PH evidence face twice the mortality risk of non-PH counterparts (RR = 2.02, 95% CI:1.70–2.40) [4]. This underscores the urgency of surveillance strategies, particularly given the frequent absence of PH symptoms until advanced stages.

### 4.1. Interpretation in Context of Existing Literature

The observed association between AVF use and PH in ESRD patients aligns with emerging mechanistic insights into hemodynamic stress and vascular remodeling. Malin et al. reported that AVF-related high-flow states may exacerbate pulmonary vascular resistance (PVR), with a 2024 analysis identifying AVF as an independent predictor of precapillary PH (OR = 2.47, 95% CI:1.56–3.89) [17]. This is directionally consistent with our pooled OR of 2.06. The relationship between AVF flow and PH severity is further supported by echocardiographic evidence linking proximal AVF location—associated with 1240 mL/min mean flow—to 16.6 mmHg higher pulmonary arterial systolic PASP compared to distal AVF [19].

Discrepancies in literature likely stem from methodological heterogeneity. For instance, the inclusion of patients with varying dialysis vintages or comorbid left ventricular dysfunction may confound hemodynamic measurements. Notably, our meta-analysis revealed a strong correlation between PAP and HD duration (r = 0.702) [18], implicating cumulative volume overload as a critical cofactor. This temporal relationship is reinforced by longitudinal data showing PH prevalence rising from 17% to 31% after six years of AVF use [12,17]. The paradoxical finding of reduced RVSP post-AVF creation in some cohorts [20] may reflect improved volume status rather than intrinsic pulmonary vascular changes, highlighting the need for timed echocardiographic assessments relative to dialysis sessions.

### 4.2. Mechanisms and Pathophysiology

The pathogenesis of AVF-associated PH in ESRD patients involves interrelated hemodynamic, vascular, and cardiac remodeling processes as shown below in Table 3:

### 4.3. Heterogeneity and Methodological Considerations

This meta-analysis shows contrasting heterogeneity patterns between primary and secondary outcomes. The primary outcome (PH prevalence) demonstrated minimal heterogeneity (Q = 5.37, *p* = 0.80; I^2^ = 0%) attributed to uniform observational study designs. Conversely, secondary analysis of mean PAP differences showed extreme heterogeneity (I^2^ = 99.4%) due to varied assessment methods. Additional variability arose from AVF characteristics, including flow rates (800–2200 mL/min) and duration of use (1–10 years), which differentially impact pulmonary hemodynamics [21].

#### 4.3.1. Analytical Approach and Sensitivity

The DerSimonian–Laird random-effects model was selected a priori to account for anticipated clinical and methodological diversity [24]. Leave-one-out sensitivity analysis indicated that the pooled odds ratio was sensitive to individual studies (OR range across iterations: 1.19–2.22): removal of most studies left the estimate broadly unchanged, but exclusion of Fabbian et al. reduced it to a non-significant OR = 1.19 (95% CI:0.81–1.75). This outlier effect underscores the disproportionate influence single studies can exert in meta-analyses with limited included trials; a phenomenon exacerbated by the DerSimonian–Laird estimator’s sensitivity to small-study effects [24].

#### 4.3.2. Publication Bias Assessment

Egger’s regression test (intercept = 0.14, *p* = 0.83) and Begg’s rank correlation (Kendall’s τ = 0.29, *p* = 0.29) found no evidence of publication bias. However, these tests have limited power in meta-analyses with fewer than 10 studies, as acknowledged in their original derivations [25,26]. The symmetrical distribution of effect sizes in the funnel plot (Figure 4) further supports the absence of small-study effects, though this visual method remains subjective in smaller syntheses [26].

### 4.4. Key Limitations

There were several limitations to our study. First, all included studies were observational and predominantly cross-sectional, precluding causal inference; unmeasured confounders—such as left ventricular dysfunction, sleep apnea, or pulmonary embolism—may partially explain the observed AVF/AVG–PH association. Second, the pooled estimate was sensitive to individual studies, losing statistical significance when a single study was removed (Table 2), so the magnitude of the association should be regarded as uncertain. Third, PH was defined heterogeneously across studies, by echocardiographic estimation of PASP/RVSP in most and by right heart catheterization in a minority, and with differing thresholds (e.g., mean PAP ≥ 20 vs. ≥25 mmHg); because only a single study used invasive measurement, a formal subgroup analysis separating invasive from non-invasive definitions was not feasible, and this remains an important source of measurement heterogeneity, particularly for the secondary outcome. The timing of pressure assessment relative to dialysis, which can alter echocardiographic estimates by several mmHg through interdialytic volume shifts, was also inconsistently reported.

Additionally, cross-sectional designs dominated the evidence base, obscuring the temporal relationship between AVF creation and PH onset. Only two studies provided longitudinal PAP data pre- and post-AVF placement. Finally, none of the studies objectively quantified AVF flow rates using Doppler ultrasonography or magnetic resonance angiography, relying instead on presumed flow differences based on fistula location.

### 4.5. Clinical Implications

Because the association between AVF/AVG use and PH observed here derives from observational data and is sensitive to individual studies, the considerations below should be viewed as hypotheses to guide future evaluation rather than as established changes to clinical practice.

#### 4.5.1. Surveillance and Monitoring

Whether echocardiographic screening for PH should be incorporated into routine care for long-term hemodialysis patients—particularly those with brachial AVFs or prolonged dialysis vintage—is a reasonable question for prospective evaluation; current evidence is insufficient to recommend it as standard practice.

#### 4.5.2. Access Planning and Modification

Proactive flow monitoring via ultrasound dilution or Doppler techniques is critical, with KDOQI guidelines recommending intervention for AVF flows < 400–500 mL/min and AVG flows < 600 mL/min [27]. In high-risk patients—such as those with preexisting left ventricular dysfunction or elevated RVSP—the relative merits of PD or catheter-based hemodialysis warrant prospective study before any conclusion can be drawn about their effect on PH risk [6,28].

#### 4.5.3. Therapeutic Interventions

Renal transplantation, where feasible, may improve pulmonary pressures in selected patients [29]. Importantly, no targeted pulmonary vasodilator therapy has been validated specifically for PH in the ESRD population, and the use of agents such as phosphodiesterase-5 inhibitors in this setting is supported only by limited observational data, with uncertain effects on clinically important outcomes [6,30]. Such treatments should therefore be regarded as investigational rather than evidence-based for access-related PH.

### 4.6. Future Research Directions

The evolving understanding of AVF/AVG-associated PH in ESRD patients calls for targeted investigations to address critical knowledge gaps. Building on current evidence, several priority areas emerge:

#### 4.6.1. Prospective Human Studies

While retrospective analyses suggest PH prevalence rises from 17% to 31% over six years of AVF use, prospective RHC data are lacking [7,18]. Standardized protocols should include baseline RHC measurements before AVF placement, followed by serial assessments at 6-month intervals. Such designs could clarify whether high-flow AVFs directly drive PH or merely unmask preexisting subclinical disease.

#### 4.6.2. Therapeutic Trials

Randomized controlled trials (RCTs) evaluating AVF flow reduction strategies are critical. Pilot data suggest banding or ligation lowers PAP by 15–30 mmHg, but evidence remains limited to case series [31]. An RCT comparing moderate-flow AVFs (500–800 mL/min) vs. standard high-flow access could determine whether flow restriction mitigates PH without compromising dialysis adequacy. Concurrently, pulmonary vasodilators such as riociguat require rigorous evaluation of safety and efficacy before any role in this population can be defined, given that current data are limited and largely observational [1,32].

#### 4.6.3. Diagnostic Standardization

Developing consensus guidelines for PH screening in ESRD is paramount. Current reliance on echocardiography introduces measurement variability due to interdialytic fluid shifts, overestimating PAP by up to 10 mmHg [3,18]. A multicenter study comparing pre- vs. post-dialysis RHC measurements could establish optimal timing for PH diagnosis.

## 5. Conclusions

This meta-analysis found a statistically significant association between AVF/AVG use and PH prevalence in dialysis patients. However, this association was derived entirely from observational studies and was sensitive to individual influential studies, and the heterogeneous PH definitions, residual confounding, and predominantly cross-sectional design preclude any causal interpretation. The findings should therefore be read as identifying a possible association in need of prospective confirmation.

Rather than supporting specific changes to clinical practice, the findings highlight questions that prospective work should address—whether PH monitoring, individualized access planning, or modification of dialysis modality or AVF flow alters outcomes in higher-risk patients. Adequately powered prospective hemodynamic studies, including baseline assessment before AVF creation, will be required to determine whether the association is causal and to inform management.

## Figures and Tables

**Table 1:** List of Included Studies with Corresponding Odds Ratios and 95% Confidence Intervals.

Study	Sample Size	Odds Ratio	95% Confidence Interval
Yigla et al. (2003) [7]	75	22.34	[1.28, 390.55]
Havlucu et al. (2007) [8]	48	1.98	[0.63, 6.26]
Abdelwhab et al. (2008) [9]	76	1.68	[0.65, 4.37]
Hemnes et al. (2010) [10]	91	1.16	[0.44, 3.08]
Unal et al. (2010) [11]	20	1.71	[0.4, 7.34]
Fabbian et al. (2011) [12]	56	6.23	[1.84, 21.12]
Ramasubbu et al. (2011) [13]	90	1.9	[0.55, 6.63]
Pabst et al. (2012) [14]	62	1.4	[0.45, 4.41]
Unal et al. (2013) [15]	50	0.84	[0.37, 1.9]
Mukhtar et al. (2014) [16]	80	3.9	[0.95, 16.01]
Malin et al. (2024) [17]	651	2.2	[1.44, 3.35]

**Table 2:** Sensitivity Analysis of the Included Studies.

Excluded Study	Pooled OR	95% Confidence Interval
Unal et al. (2013) [15]	1.74	[0.97–3.11]
Mukhtar et al. (2014) [16]	2.09	[1.78–2.45]
Hemnes et al. (2010) [10]	2.09	[1.68–2.61]
Abdelwhab et al. (2008) [9]	1.97	[1.45–2.67]
Fabbian et al. (2011) [12]	1.19	[0.81–1.75]
Havlucu et al. (2007) [8]	1.93	[1.41–2.65]
Pabst et al. (2012) [14]	1.99	[1.48–2.67]
Ramasubbu et al. (2011) [13]	1.94	[1.41–2.65]
Unal et al. (2010) [11]	1.94	[1.42–2.66]
Malin et al. (2024) [17]	1.72	[1.16–2.55]

**Table 3:** Proposed Mechanisms of AVF-Associated Pulmonary Hypertension and Supporting Evidence.

Proposed Mechanism	Supporting Evidence
High-flow AVF → ↑ cardiac output → pulmonary vascular congestion	AVF flow > 1500 mL/min correlates with PH prevalence (OR = 2.47) [17], while flow reduction via ligation reverses left ventricular hypertrophy (ΔLV mass: −22.1 g) [21]. Elevated cardiac output (6.4 L/min in PH patients) overwhelms pulmonary capacitance [22].
Uremia-induced endothelial dysfunction → ↑PVR	ESRD-related oxidative stress reduces nitric oxide bioavailability, exacerbating endothelin-1-mediated vasoconstriction (r = 0.93 between PAP and PVR) [6,23].
Chronic volume overload → right ventricular strain	Prolonged hemodialysis (>6 years) amplifies PAP elevation (r = 0.702) [17], while interdialytic fluid shifts confound echocardiographic RVSP measurements [20].
AVF flow duration → cumulative vascular remodeling	Muscularization of distal pulmonary arteries occurs in high-flow animal models, mirrored by PH prevalence rising from 17% to 31% over six years of AVF use [17,22].

## Data Availability

Data sharing is not applicable to this article as no datasets were generated or analyzed during the current study.

## Supplementary Materials

The following supporting information can be downloaded at: https://www.mdpi.com/article/10.3390/arm94040046/s1, PRISMA Cheklist and The detailed Newcastle–Ottawa Scale (NOS) scoring for each included study is provided in Supplementary Material.
