# Supplementary material for: Prevalence and Impact of Pulmonary Hypertension Associated with Arteriovenous Fistulas and Grafts in End-Stage Renal Disease: A Systematic Review and Meta-Analysis
Source: Adv Respir Med. 2026 Jul 6;94(4):46. doi: 10.3390/arm94040046 (PMC13397879; doi:10.3390/arm94040046)
Supplement: Supplementary file 1 [file arm-94-00046-s001.zip › arm-4356174-supplementary.pdf]

PRISMA 2020 Checklist

Section and Topic	Item #	Checklist item	Location where item is reported
**TITLE**			
Title	1	Identify the report as a systematic review.	Title (Page 1): "…A Systematic Review and Meta-analysis".
**ABSTRACT**			
Abstract	2	See the PRISMA 2020 for Abstracts checklist.	Abstract (Page 1): structured into background/objectives, methods, results, and conclusions per the PRISMA 2020 for Abstracts checklist.
**INTRODUCTION**			
Rationale	3	Describe the rationale for the review in the context of existing knowledge.	Introduction (§1, paragraphs 1–3): existing prevalence estimates (17–70%), pathophysiology of high-flow AVF, modality differences, and the twofold mortality association in ESRD-PH.
Objectives	4	Provide an explicit statement of the objective(s) or question(s) the review addresses.	Introduction (§1, final paragraph): "…this meta-analysis systematically quantifies the relationship between AVF/AVG use and PH prevalence in ESRD populations, while exploring underlying sources of heterogeneity."
**METHODS**			
Eligibility criteria	5	Specify the inclusion and exclusion criteria for the review and how studies were grouped for the syntheses.	Methods §2.2 (Eligibility Criteria): inclusion (PICO — adults ≥18 with ESRD on dialysis; AVF/AVG vs. non-AVF; PH prevalence or mean PAP outcomes; observational studies and RCTs) and exclusion criteria explicitly listed.
Information sources	6	Specify all databases, registers, websites, organisations, reference lists and other sources searched or consulted to identify studies. Specify the date when each source was last searched or consulted.	Methods §2.1 (Search Strategy): PubMed, Embase, Scopus, and Web of Science searched from database inception through 31 December 2024; reference lists of included studies and relevant systematic reviews were also hand-searched.
Search strategy	7	Present the full search strategies for all databases, registers and websites, including any filters and limits used.	Methods §2.1 (Search Strategy): full MeSH/Boolean string provided — ("Renal Dialysis"[MeSH] OR "End-Stage Renal Disease"[MeSH] OR "ESRD") AND ("Arteriovenous Fistula"[MeSH] OR "AVF" OR "Vascular Access") AND ("Hypertension, Pulmonary"[MeSH] OR "Pulmonary Hypertension" OR "Pulmonary Artery Pressure" OR

Section and Topic	Item #	Checklist item	Location where item is reported
			"RVSP" OR "PASP"). No language or date restrictions applied.
Selection process	8	Specify the methods used to decide whether a study met the inclusion criteria of the review, including how many reviewers screened each record and each report retrieved, whether they worked independently, and if applicable, details of automation tools used in the process.	Methods §2.1 and §2.3: citations imported into Covidence; duplicates removed; titles/abstracts then full texts screened by three reviewers (MA, SB, EB) independently, with discrepancies adjudicated by a fourth reviewer (GK).
Data collection process	9	Specify the methods used to collect data from reports, including how many reviewers collected data from each report, whether they worked independently, any processes for obtaining or confirming data from study investigators, and if applicable, details of automation tools used in the process.	Methods §2.3 (Data Extraction and Quality Assessment): three reviewers (MA, SB, EB) independently extracted data using a pre-defined, standardized data collection form; discrepancies resolved by consensus or by adjudication from a fourth reviewer (GK). No automation tools were used. Original investigators were not contacted.
Data items	10a	List and define all outcomes for which data were sought. Specify whether all results that were compatible with each outcome domain in each study were sought (e.g. for all measures, time points, analyses), and if not, the methods used to decide which results to collect.	Methods §2.3 and §2.4: primary outcome — odds of PH (AVF/AVG vs. non-AVF); secondary outcome — mean difference in pulmonary artery pressure (PAP, mPAP, PASP, or RVSP). All compatible PH definitions and assessment time points reported by each study were extracted.
	10b	List and define all other variables for which data were sought (e.g. participant and intervention characteristics, funding sources). Describe any assumptions made about any missing or unclear information.	Methods §2.3: extracted variables included first author, year, study design, patient population characteristics, total sample size, number of patients per access group, definition and diagnostic method of PH (echocardiography vs. RHC), and the number of PH cases in each access group. Where PH was reported only as a percentage, absolute numbers were back-calculated from the corresponding sample size.
Study risk of bias assessment	11	Specify the methods used to assess risk of bias in the included studies, including details of the tool(s) used, how many reviewers assessed each study and whether they worked independently, and if applicable, details of automation tools used in the process.	Methods §2.3: risk of bias assessed with the Newcastle–Ottawa Scale (NOS) for observational studies across three domains (selection, comparability, outcome); Cochrane RoB 2.0 was reserved for any RCTs. Two reviewers assessed each study

PRISMA 2020 Checklist

Section and Topic	Item #	Checklist item	Location where item is reported
			independently; discrepancies adjudicated by a fourth reviewer.
Effect measures	12	Specify for each outcome the effect measure(s) (e.g. risk ratio, mean difference) used in the synthesis or presentation of results.	Methods §2.4 (Statistical Analysis): primary outcome — odds ratio (OR) with 95% CI from 2×2 contingency tables; secondary outcome — mean difference in PAP with 95% CI.
Synthesis methods	13a	Describe the processes used to decide which studies were eligible for each synthesis (e.g. tabulating the study intervention characteristics and comparing against the planned groups for each synthesis (item #5)).	Methods §2.2 and §2.4: studies eligible for the primary synthesis required dichotomous PH data in AVF/AVG vs. non-AVF groups; the secondary synthesis included studies reporting continuous PAP values for both groups.
	13b	Describe any methods required to prepare the data for presentation or synthesis, such as handling of missing summary statistics, or data conversions.	Methods §2.4 and Results §3.3: log-transformed ORs and standard errors used for pooling; one study (Yigla et al.) with a zero-event control cell was excluded a priori from the pooled estimate due to extreme variance.
	13c	Describe any methods used to tabulate or visually display results of individual studies and syntheses.	Methods §2.4 and Results §3: forest plot (Figure 2), study-level summary table (Table 1), sensitivity analysis table (Table 2), Egger's plot (Figure 3), and Begg's plot (Figure 4).
	13d	Describe any methods used to synthesize results and provide a rationale for the choice(s). If meta-analysis was performed, describe the model(s), method(s) to identify the presence and extent of statistical heterogeneity, and software package(s) used.	Methods §2.4: DerSimonian–Laird random-effects model selected a priori to account for between-study heterogeneity; heterogeneity quantified by Cochran's Q and I^2 with conventional cutoffs (0–30% low; 30–50% moderate; 50–75% substantial; >75% considerable). Analyses performed in Python 3.8 using statsmodels, scipy, and matplotlib.
	13e	Describe any methods used to explore possible causes of heterogeneity among study results (e.g. subgroup analysis, meta-regression).	Discussion §4.3 (Heterogeneity and Methodological Considerations): variability discussed in relation to AVF flow rates (800–2,200 mL/min), dialysis vintage (1–10 years), and assessment modality (echocardiography vs. RHC). Formal subgroup analysis or meta-regression was not feasible given k = 10.
	13f	Describe any sensitivity analyses conducted to assess robustness of the synthesized results.	Methods §2.4 and Results §3.4: pre-specified leave-one-out sensitivity

PRISMA 2020 Checklist

Section and Topic	Item #	Checklist item	Location where item is reported
			analysis (results presented in Table 2).
Reporting bias assessment	14	Describe any methods used to assess risk of bias due to missing results in a synthesis (arising from reporting biases).	Methods §2.4: Egger's regression test and Begg's rank-correlation (Kendall's τ) used together to address the limited power of either test alone given k = 10; visual funnel-plot inspection was not used as a stand-alone method.
Certainty assessment	15	Describe any methods used to assess certainty (or confidence) in the body of evidence for an outcome.	Methods §2.3 and Discussion §4.3–§4.4: certainty appraised narratively via NOS-based quality ratings (most studies moderate quality) together with explicit discussion of risk of bias, heterogeneity, and indirectness. A formal GRADE summary was not produced owing to the observational, predominantly cross-sectional evidence base — acknowledged in §4.4.
**RESULTS**			
Study selection	16a	Describe the results of the search and selection process, from the number of records identified in the search to the number of studies included in the review, ideally using a flow diagram.	Results §3.1 (Study Selection and Characteristics) and Figure 1 (PRISMA flow diagram). Ten observational studies including 1,199 dialysis patients were retained.
	16b	Cite studies that might appear to meet the inclusion criteria, but which were excluded, and explain why they were excluded.	Results §3.3 (Primary Outcome): Yigla et al. (2003) was excluded from the pooled estimate due to a zero-event count in the control arm yielding an OR of 22.34 with a 95% CI of [1.28–390.55] — unacceptable variance for inclusion in the pooled model. The study is still listed in Table 1 for transparency.
Study characteristics	17	Cite each included study and present its characteristics.	Results §3.1 and Table 1 (List of Included Studies with Corresponding Odds Ratios and 95% Confidence Intervals).
Risk of bias in studies	18	Present assessments of risk of bias for each included study.	Results §3.2 (Quality Assessment): NOS scores ranged 5–7 out of 9, with moderate methodological quality predominating. Full per-study NOS scoring is provided in Supplementary Material 1.
Results of individual studies	19	For all outcomes, present, for each study: (a) summary statistics for each group (where appropriate) and (b) an effect estimate and its precision (e.g. confidence/credible interval), ideally using structured tables or plots.	Table 1 (study-level sample sizes, ORs, and 95% CIs) and Figure 2

PRISMA 2020 Checklist

Section and Topic	Item #	Checklist item	Location where item is reported
			(forest plot).
Results of syntheses	20a	For each synthesis, briefly summarise the characteristics and risk of bias among contributing studies.	Results §3.1–§3.2: contributing studies were exclusively observational (cross-sectional and cohort), predominantly of moderate methodological quality, and used predominantly echocardiographic PH definitions.
	20b	Present results of all statistical syntheses conducted. If meta-analysis was done, present for each the summary estimate and its precision (e.g. confidence/credible interval) and measures of statistical heterogeneity. If comparing groups, describe the direction of the effect.	Results §3.3 and Figure 2: pooled OR = 2.06 (95% CI: 1.69–2.52); Q = 5.37, df = 9, p = 0.80; $I^2 = 0\%$. Results §3.6 (secondary analysis): pooled mean PAP difference = 5.55 mmHg (95% CI: 0.63–10.48); $I^2 = 99.4\%$.
	20c	Present results of all investigations of possible causes of heterogeneity among study results.	Discussion §4.3 (Heterogeneity and Methodological Considerations): contrasting heterogeneity patterns between the primary ($I^2 = 0\%$) and secondary ($I^2 = 99.4\%$) outcomes explained by uniformity of dichotomous outcome reporting vs. variability in continuous PAP measurement methodology, AVF flow characteristics, and dialysis vintage.
	20d	Present results of all sensitivity analyses conducted to assess the robustness of the synthesized results.	Results §3.4 and Table 2 (Leave-One-Out Sensitivity Analysis): OR remained ≥ 1.72 across all leave-one-out iterations except when Fabbian et al. was removed (OR 1.19; 95% CI 0.81–1.75).
Reporting biases	21	Present assessments of risk of bias due to missing results (arising from reporting biases) for each synthesis assessed.	Results §3.5 (Publication Bias): Egger's regression intercept = 0.14 (p = 0.83); Begg's Kendall's $\tau = 0.29$ (p = 0.29). Results displayed in Figure 3 and Figure 4. No evidence of publication bias.
Certainty of evidence	22	Present assessments of certainty (or confidence) in the body of evidence for each outcome assessed.	Results §3.2 and Discussion §4.3–§4.4: certainty is downgraded narratively because of the observational, predominantly cross-sectional evidence base, moderate NOS quality, and substantial heterogeneity in the secondary continuous outcome. No formal GRADE summary table is provided

PRISMA

PRISMA 2020 Checklist

Section and Topic	Item #	Checklist item	Location where item is reported
			(acknowledged in §4.4).
**DISCUSSION**			
Discussion	23a	Provide a general interpretation of the results in the context of other evidence.	Discussion §4 (opening paragraphs) and §4.1 (Interpretation in Context of Existing Literature): findings compared with Malin et al. 2024 (OR 2.47 for precapillary PH) and longitudinal data showing PH prevalence rising from 17% to 31% over six years.
	23b	Discuss any limitations of the evidence included in the review.	Discussion §4.4 (Key Limitations): observational designs, unmeasured confounders (LV dysfunction, sleep apnea, PE), variable PH definitions (echocardiography vs. RHC; ≥ 20 vs. ≥ 25 mmHg cutoffs), cross-sectional dominance, and lack of objective AVF flow quantification.
	23c	Discuss any limitations of the review processes used.	Discussion §4.3–§4.4: small number of contributing studies (k = 10) limits power of Egger's/Begg's tests and precludes formal meta-regression; secondary continuous synthesis hampered by extreme heterogeneity (I^2 = 99.4%); no formal GRADE certainty rating was performed.
	23d	Discuss implications of the results for practice, policy, and future research.	Discussion §4.5 (Clinical Implications) — surveillance, access planning, and therapeutic interventions — and §4.6 (Future Research Directions) — prospective RHC studies, RCTs of AVF flow reduction, and diagnostic standardisation.
**OTHER INFORMATION**			
Registration and protocol	24a	Provide registration information for the review, including register name and registration number, or state that the review was not registered.	Methods §2 (opening paragraph): registered prospectively in PROSPERO, registration number CRD420251051561.
	24b	Indicate where the review protocol can be accessed, or state that a protocol was not prepared.	Methods §2: protocol accessible via the PROSPERO record (CRD420251051561) at https://www.crd.york.ac.uk/prospero/.
	24c	Describe and explain any amendments to information provided at registration or in the protocol.	No amendments were made to the registered protocol.
Support	25	Describe sources of financial or non-financial support for the review, and the role of the funders or sponsors in the review.	Back matter — Funding statement: "The authors received no financial

PRISMA 2020 Checklist

Section and Topic	Item #	Checklist item	Location where item is reported
			support for the research, authorship, and/or publication of this article." No sponsor or funder had any role in the review.
Competing interests	26	Declare any competing interests of review authors.	Back matter — Conflicts of Interest: "The authors declare that they have no competing interests to report."
Availability of data, code and other materials	27	Report which of the following are publicly available and where they can be found: template data collection forms; data extracted from included studies; data used for all analyses; analytic code; any other materials used in the review.	Back matter — Data Availability Statement: all data extracted from the included studies are available within the original publications cited in the References. The standardized data-extraction template, the per-study NOS quality-assessment ratings, and the Python analytic code (statsmodels, scipy, matplotlib) are available from the corresponding author on reasonable request.

From: Page MJ, McKenzie JE, Bossuyt PM, Boutron I, Hoffmann TC, Mulrow CD, et al. The PRISMA 2020 statement: an updated guideline for reporting systematic reviews. BMJ 2021;372:n71. doi: 10.1136/bmj.n71. This work is licensed under CC BY 4.0. To view a copy of this license, visit https://creativecommons.org/licenses/by/4.0/

Study	Selection (max 4)	Comparability (max 2)	Outcome (max 3)	Total NOS Score (/9)
Unal et al. (2013) [15]	4	1	2	7
Mukhtar et al. (2014)	3	1	2	6
Hemnes et al. (2010)	3	1	2	6
Abdelwhab & Elshinnawy (2008)	3	1	2	6
Fabbian et al. (2010)	3	2	2	7
Havlucu et al. (2007)	4	1	2	7
Pabst et al. (2012)	3	1	2	6
Ramasubbu et al. (2010)	2	1	2	5
Tasdemir et al. (2010)	2	1	3	6
Malin et al. (2024)	3	1	2	6

Domain	Item (Max Stars)	Assessment for Abdelwhab & Elshinnawy (2008)	Stars
**Selection**	1. Representativeness of the exposed cohort (HD with AVF)	ESRD patients on regular HD with AVF recruited from two university hospitals; consecutive and representative	★
	2. Selection of the non-exposed cohort (conservative management)	ESRD patients on conservative (non-dialysis) management from same setting; appropriate comparator group	★
	3. Ascertainment of exposure (AVF)	AVF status confirmed by clinical records and Doppler ultrasound flow measurements	★
	4. Demonstration that outcome (PH) not present at start	No baseline echocardiography prior to AVF/HD initiation; cross-sectional design	
**Comparability**	1. Comparability of cohorts on basis of design or analysis (control for confounders)	Groups matched for age, gender, and key comorbidities; exclusion of major cardiac/pulmonary diseases	★
	2. Additional comparability (adjustment for other confounders)	No multivariate adjustment for all potential confounders (e.g., LV diastolic dysfunction, dialysis vintage)	
**Outcome**	1. Assessment of outcome (PH diagnosis)	PH assessed by standardized Doppler echocardiography at dry weight, blinded interpretation	★
	2. Was follow-up long enough for	Cross-sectional design; no longitudinal follow-	

Domain	Item (Max Stars)	Assessment for Abdelwhab & Elshinnawy (2008)	Stars
	outcomes to occur?	up	
	3. Adequacy of follow-up of cohorts	All enrolled patients completed assessment; no loss to follow-up	★

Domain	Item (Max Stars)	Assessment for Fabbian et al. (2010)	Stars
**Selection**	1. Representativeness of the exposed cohort (HD with AVF)	Consecutive ESRD patients on HD with AVF from a single center; representative and well-described cohort	★
	2. Selection of the non-exposed cohort (PD patients)	PD patients from the same center and timeframe used as comparator group; appropriate and well-matched controls	★
	3. Ascertainment of exposure (AVF)	AVF status and dialysis modality confirmed by clinical records	★
	4. Demonstration that outcome (PH) not present at start	Cross-sectional design; no baseline PH assessment before HD/AVF initiation	
**Comparability**	1. Comparability of cohorts on basis of design or analysis (control for confounders)	Groups matched for age, sex, and major comorbidities; exclusion of significant lung/cardiac disease	★
	2. Additional comparability (adjustment for other confounders)	Multivariate logistic regression used to adjust for dialysis vintage, blood pressure, ejection fraction, and diabetes	★

Domain	Item (Max Stars)	Assessment for Fabbian et al. (2010)	Stars
**Outcome**	1. Assessment of outcome (PH diagnosis)	PH assessed by standardized, blinded echocardiography at dry weight; validated and reproducible method	★
	2. Was follow-up long enough for outcomes to occur?	Cross-sectional design; no longitudinal follow-up for incident PH	
	3. Adequacy of follow-up of cohorts	All 56 eligible patients completed assessment; no loss to follow-up	★

Domain	Item (Max Stars)	Assessment for Havlucu et al. (2007)	Stars
**Selection**	1. Representativeness of the exposed cohort (HD with AVF)	Consecutive ESRD patients on HD with AVF from a university hospital; well-described and representative cohort	★
	2. Selection of the non-exposed cohort (predialysis CRF patients)	Predialysis CRF patients from the same setting and timeframe; appropriate and well-matched controls	★
	3. Ascertainment of exposure (AVF)	AVF status and dialysis modality confirmed by clinical records and Doppler sonography	★
	4. Demonstration that outcome (PH) not present at start	Excluded patients with pre-existing pulmonary or cardiac disease; baseline echocardiography performed before HD/AVF	★
**Comparability**	1. Comparability of cohorts on basis of design or analysis (control for confounders)	Groups matched for age, sex, comorbidities; strict exclusion of confounders (lung/cardiac disease, smoking, etc.)	★
	2. Additional comparability (adjustment for other confounders)	No multivariate statistical adjustment for all potential confounders (e.g., dialysis vintage, LV function, mineral metabolism)	

Domain	Item (Max Stars)	Assessment for Havlucu et al. (2007)	Stars
**Outcome**	1. Assessment of outcome (PH diagnosis)	PH assessed by standardized, blinded Doppler echocardiography at dry weight; validated and reproducible method	★
	2. Was follow-up long enough for outcomes to occur?	Mean follow-up 7.5 months; some longitudinal data but limited for chronic PH development	
	3. Adequacy of follow-up of cohorts	All 48 eligible patients completed follow-up; no loss to follow-up	★

Domain	Item (Max Stars)	Assessment for Hemnes et al. (2010)	Stars
**Selection**	1. Representativeness of the exposed cohort (AVF/AVG patients)	Patients were all ESRD patients on maintenance hemodialysis at two urban centers; inclusion was comprehensive and consecutive	★
	2. Selection of the non-exposed cohort (catheter-based HD)	Catheter-based HD patients included as a comparator group from the same centers and timeframe	★
	3. Ascertainment of exposure (vascular access type)	Vascular access type (AVF/AVG vs. catheter) was well-documented in dialysis records	★
	4. Demonstration that outcome (PH) not present at start	Cross-sectional design; no baseline PH assessment prior to HD initiation or access placement	
**Comparability**	1. Comparability of cohorts on basis of design or analysis (control for confounders)	Multivariate analysis adjusted for age, gender, and ethnicity; groups compared for major comorbidities	★

Domain	Item (Max Stars)	Assessment for Hemnes et al. (2010)	Stars
	2. Additional comparability (adjustment for other confounders)	No further multivariate adjustment for all potential confounders (e.g., LV function, dialysis vintage)	
**Outcome**	1. Assessment of outcome (PH diagnosis)	PH defined by standardized echocardiographic criteria (RVSP ≥45 mmHg); echocardiography performed at a single institution	★
	2. Was follow-up long enough for outcomes to occur?	Cross-sectional; no follow-up for incident PH or progression	
	3. Adequacy of follow-up of cohorts	All 91 eligible patients included; no loss to follow-up	★

Domain	Item (Max Stars)	Assessment for Malin et al. (2024)	Stars
**Selection**	1. Representativeness of the exposed cohort (CKD/ESRD with AVF)	Large, consecutive cohort of CKD stage 4/5 patients with AVF from a single academic center; well-defined and representative	★
	2. Selection of the non-exposed cohort (CKD/ESRD without AVF)	Non-AVF group included CKD 4/5 patients from the same center and period, with and without dialysis; appropriate comparator	★
	3. Ascertainment of exposure (AVF status)	AVF status confirmed by clinical records and dialysis history	★
	4. Demonstration that outcome (PH) not present at start	Cross-sectional design; no baseline PH assessment before AVF creation or dialysis initiation	

Domain	Item (Max Stars)	Assessment for Malin et al. (2024)	Stars
**Comparability**	1. Comparability of cohorts on basis of design or analysis (control for confounders)	Multivariable logistic regression adjusted for age, sex, lung disease, sleep apnea, connective tissue disease, anemia, heart failure	★
	2. Additional comparability (adjustment for other confounders)	Further relevant confounders (e.g., dialysis vintage, AVF flow, residual renal function) not fully adjusted	
**Outcome**	1. Assessment of outcome (PH diagnosis)	PH and subtypes diagnosed by right heart catheterization (gold standard), using standardized, blinded criteria	★
	2. Was follow-up long enough for outcomes to occur?	Cross-sectional design; no longitudinal follow-up for incident PH or progression	
	3. Adequacy of follow-up of cohorts	All 651 eligible patients included; no loss to follow-up reported	★

Domain	Item (Max Stars)	Assessment for Mukhtar et al. (2014)	Stars
**Selection**	1. Representativeness of the exposed cohort (AVF patients)	ESRD patients on maintenance HD, mostly with AVF, recruited consecutively from a single nephrology center; reasonably representative	★
	2. Selection of the non-exposed cohort (controls: tunneled catheter, AVG)	Included patients with tunneled catheters and one with AV graft as comparison; groups defined but non-AVF group small	★
	3. Ascertainment of exposure (AVF/vascular access)	Vascular access type documented from clinical records	★
	4. Demonstration that outcome (PH)	No baseline echocardiography before HD initiation;	

Domain	Item (Max Stars)	Assessment for Mukhtar et al. (2014)	Stars
	not present at start	unclear if PH was present prior to AVF/HD	
**Comparability**	1. Comparability of cohorts on basis of design or analysis (control for confounders)	Exclusion criteria for major confounders (lung disease, valve disease, sleep apnea); groups compared for age/gender	★
	2. Additional comparability (adjustment for other confounders)	No multivariate adjustment for other confounders (e.g., LV function, comorbidities, dialysis vintage)	
**Outcome**	1. Assessment of outcome (PH diagnosis)	PH assessed by echocardiography, performed post-dialysis at dry weight by a cardiologist; standardized and blinded assessment	★
	2. Was follow-up long enough for outcomes to occur?	Cross-sectional design; no follow-up for PH progression or incident cases	
	3. Adequacy of follow-up of cohorts	All 80 enrolled patients completed assessment; no loss to follow-up	★

Domain	Item (Max Stars)	Assessment for Pabst et al. (2012)	Stars
**Selection**	1. Representativeness of the exposed cohort (dialysis patients)	Consecutive, symptomatic ESRD patients (WHO FC ≥ II) on hemodialysis at a university center; well-defined but selected for dyspnea (may limit generalizability)	★
	2. Selection of the non-exposed cohort (CKD without dialysis)	CKD stage 4–5 patients with similar inclusion/exclusion criteria, from same center and time period; appropriate comparator	★

Domain	Item (Max Stars)	Assessment for Pabst et al. (2012)	Stars
	3. Ascertainment of exposure (dialysis status)	Dialysis status confirmed by clinical records and treatment history	★
	4. Demonstration that outcome (PH) not present at start	Cross-sectional design; no baseline PH assessment prior to dialysis initiation; all patients symptomatic at enrollment	
**Comparability**	1. Comparability of cohorts on basis of design or analysis (control for confounders)	Groups matched for key comorbidities; exclusion of major confounders (LV dysfunction, significant valve disease, severe COPD, anemia, malignancy, etc.)	★
	2. Additional comparability (adjustment for other confounders)	No multivariate statistical adjustment for all potential confounders (e.g., age, BMI, time since CKD diagnosis)	
**Outcome**	1. Assessment of outcome (PH diagnosis)	PH diagnosed by right heart catheterization (gold standard), using standardized criteria and blinded assessment	★
	2. Was follow-up long enough for outcomes to occur?	Cross-sectional design; no longitudinal follow-up for incident PH	
	3. Adequacy of follow-up of cohorts	All 62 eligible patients completed assessment; no loss to follow-up	★

Domain	Item (Max Stars)	Assessment for Ramasubbu et al. (2010)	Stars
**Selection**	1. Representativeness of the exposed cohort (HD patients)	Consecutive, unselected chronic hemodialysis patients from a single US center; representative of the local HD population	★
	2. Selection of the non-exposed cohort (non-AVF/AVG access)	All access types included (AVF, AVG, catheter), but no explicit non-dialysis or pre-dialysis control group	

Domain	Item (Max Stars)	Assessment for Ramasubbu et al. (2010)	Stars
	3. Ascertainment of exposure (vascular access type)	Access type (AVF, AVG, catheter) documented from clinical records	★
	4. Demonstration that outcome (PH) not present at start	Cross-sectional design; no baseline PH assessment prior to HD or access placement	
**Comparability**	1. Comparability of cohorts on basis of design or analysis (control for confounders)	Groups compared for age, gender, comorbidities; exclusion of major confounders (e.g., significant valve disease, severe LV dysfunction)	★
	2. Additional comparability (adjustment for other confounders)	No multivariate adjustment for all relevant confounders (e.g., dialysis vintage, access type, volume status)	
**Outcome**	1. Assessment of outcome (PH diagnosis)	PH assessed by standardized, blinded echocardiography using prespecified TR jet velocity criteria	★
	2. Was follow-up long enough for outcomes to occur?	12-month follow-up for mortality and hospitalization, but PH assessment is cross-sectional	★
	3. Adequacy of follow-up of cohorts	All 90 enrolled patients accounted for in survival analysis; minimal loss to follow-up	★

Domain	Item (Max Stars)	Assessment for Tasdemir et al. (2010)	Stars
**Selection**	1. Representativeness of the exposed cohort (AVF patients)	Patients with ESRD scheduled for AVF creation at a single center; consecutive and well-defined but small final sample (n=20)	★
	2. Selection of the non-exposed cohort (pre-AVF, pre-dialysis)	Each patient served as their own control (before and after AVF creation); no external comparator group	

Domain	Item (Max Stars)	Assessment for Tasdemir et al. (2010)	Stars
	3. Ascertainment of exposure (AVF creation)	AVF creation and exposure status confirmed by surgical and clinical records	★
	4. Demonstration that outcome (PH) not present at start	Baseline echocardiography performed before AVF creation; PH status documented pre-exposure	★
**Comparability**	1. Comparability of cohorts on basis of design or analysis (control for confounders)	Self-controlled design eliminates between-patient confounding; exclusion of major cardiac/pulmonary disease	★
	2. Additional comparability (adjustment for other confounders)	No multivariate adjustment for time-varying confounders (e.g., EPO use, nutrition, dialysis status)	
**Outcome**	1. Assessment of outcome (PH diagnosis)	PH assessed by standardized, blinded echocardiography at both time points; validated method	★
	2. Was follow-up long enough for outcomes to occur?	Mean follow-up of ~2 years (23.5 months); adequate for observing PH changes post-AVF	★
	3. Adequacy of follow-up of cohorts	Only 20 of 50 initial patients completed follow-up (40% retention); reasons for exclusion/loss to follow-up described	
